# Pediatric Tuberculosis Research and Development: Progress, Priorities and Funding Opportunities

**DOI:** 10.3390/pathogens11020128

**Published:** 2022-01-21

**Authors:** Lindsay McKenna, Ani Herna Sari, Sushant Mane, Anna Scardigli, Grania Brigden, Vanessa Rouzier, Mercedes C. Becerra, Anneke C. Hesseling, Farhana Amanullah

**Affiliations:** 1 Treatment Action Group, TB Project, New York, NY 10004, USA; 2 Global TB Community Advisory Board (TB CAB), Social Science Student at Airlangga University, Surabaya 60286, Indonesia; anihernasari@gmail.com; 3 State Pediatric Centre of Excellence for Tuberculosis, Department of Pediatrics, Grant Government Medical College, Sir J.J. Group of Hospitals, Mumbai 400008, India; drsush2006@gmail.com; 4 The Global Fund to Fight AIDS, Tuberculosis and Malaria, 1218 Geneva, Switzerland; Anna.Scardigli@theglobalfund.org (A.S.); Grania.Brigden@theglobalfund.org (G.B.); 5 Department of Pediatrics, GHESKIO Centers, Port-au-Prince HT-6110, Haiti; vrouzier@gheskio.org; 6 Department of Global Health and Social Medicine, Harvard Medical School, Boston, MA 02115, USA; Mercedes_Becerra@hms.harvard.edu; 7 Desmond Tutu TB Centre, Stellenbosch University, Cape Town Stellenbosch 7600, South Africa; annekeh@sun.ac.za; 8 Department of Pediatrics, The Indus Hospital and Health Network, The Aga Khan University Hospital, Karachi 75500, Pakistan; farhana.amanullah@tih.org.pk

**Keywords:** R&D, research and development, pediatric TB, innovation

## Abstract

In this article, we highlight technological pediatric TB research advances across the TB care cascade; discuss recently completed or ongoing work in adults and corresponding significant research gaps for children; and offer recommendations and opportunities to increase investments and accelerate pediatric TB R&D.

## 1. Introduction

Global and national health actors spent the first decade of the 21st century ignoring children affected by tuberculosis (TB)—children were not viewed as driving the TB epidemic so were not prioritized in the global TB response. The World Health Organization (WHO) only started reporting pediatric-specific (<15 years) TB disease estimates in 2012. We now know that children represent 12 percent of the global TB burden (1.2 million of 10 million) and 16% of TB deaths (230,000 of 1.5 million in 2020) each year.

If the beginning of this century is synonymous with the Middle Ages, the last ten years should be considered a renaissance period given the rapid rise in attention, effort, advocacy, and investment focused on pediatric TB. This is especially true for pediatric TB research and development (R&D), with the introduction of more sensitive and child-friendly screening and diagnostic tools, new pediatric formulations of first- and second-line drugs, and shorter treatment regimens for TB prevention and for children with non-severe drug-susceptible TB, children with drug resistant TB, and children with TB meningitis (see Table 1). The shift in thinking of children as epidemiologically insignificant to sentinels of ongoing TB transmission in households and communities is apparent. Significant progress has been made despite limited resources for pediatric TB R&D relative to both total investments in TB research and estimated research funding needs.

In 2020, global investments in pediatric TB R&D reached USD 91 million, a 56% increase from 2019, accounting for 10% of overall spending on TB R&D in 2020 [1]. Annual investments in pediatric TB R&D have enjoyed an upward trend since 2015, when Treatment Action Group (TAG) first started tracking investments in pediatric TB R&D. In 2020, expenditures on pediatric TB R&D for the first time reached a level in proportion to the burden of TB disease among children. Still, the amount spent on pediatric TB R&D is well below what is needed and far from the aspirational USD 200 million, a target set by applying the proportion of the global burden of TB morbidity and mortality among children (10%) to the USD 2 billion overall TB R&D funding ask called for at the UN High-Level Meeting on TB [2].

In this article, we highlight technological pediatric TB research advances across the TB care cascade; discuss recently completed or ongoing work in adults and corresponding significant research gaps for children; and offer recommendations and opportunities to increase investments and accelerate pediatric TB R&D.

## 2. Prevention

Younger children are at an increased risk of progressing from TB infection to active disease and of developing severe forms of TB (e.g., meningitis and miliary or other forms of disseminated disease). This increased risk underscores the importance of TB prevention measures, including contact investigations, TB preventive treatment (TPT), and TB vaccines for children and young, malnourished, and immunocompromised children in particular.

### 2.1. TB Preventive Treatment (TPT)

Isoniazid preventive therapy (IPT) has been recommended by the WHO since 1993 [3]. TPT regimens have since evolved from 6 to 36 months of daily isoniazid (IPT) to short-course rifamycin-based regimens, including 3 months of daily isoniazid and rifampicin (3HR), 4 months of daily rifampicin (4R), 3 months of once weekly isoniazid and rifapentine (3HP), and 1 month of daily isoniazid and rifapentine (1HP) [4]. Short-course, rifamycin-based TPT regimens have demonstrated greater safety, improved tolerability, and better adherence and treatment completion rates compared to IPT [5,6,7]. Ongoing and planned TPT studies are primarily focused on filling research gaps for short-course rifapentine-based regimens, including in children and adolescents. Currently, 3HP is recommended for children two years and older and 1HP is recommended for adolescents 13 years and older [4].

In Tuberculosis Trials Consortium (TBTC) Study 26 (PREVENT TB), 3HP demonstrated non-inferiority to IPT for the prevention of TB among adults, adolescents, and children as young as two years old [8]. TBTC Study 35 is a pediatric pharmacokinetic (PK) and safety study currently enrolling children with and without HIV in South Africa. Data from Study 35 will enable an extension of recommendations for, and the opportunity to benefit from access to, 3HP to children younger than 2 years old. In the AIDS Clinical Trials Group (ACTG) study A5279 (BRIEF TB), 1HP demonstrated non-inferiority to IPT for the prevention of TB among adults and adolescents (13 years and older) living with HIV [7]. An International Maternal Pediatric Adolescents AIDS Clinical Trials (IMPAACT) network study, P2024 (protocol is in development), will evaluate the PK, safety, tolerability, and acceptability of 1HP in children with and without HIV, with the intention of extending recommendations for, and the opportunity to benefit from access to, 1HP to children below 13 years of age. The results of TBTC Study 26 were first published in 2011; 10 years later, children still do not have access to short-course, rifapentine-based TPT. This is in part because of pediatric dosing and safety data gaps, but also because there are still no commercially available pediatric formulations of rifapentine available outside of clinical trials. This is despite the WHO Prequalification Program and Global Fund Expert Review Panel listing a 150 mg scored dispersible tablet of rifapentine as an urgent priority formulation since 2019, given its ability to support pediatric dosing across rifapentine-containing regimens and indications [9,10].

There are several phase 3 cluster-randomized household-based trials of TPT for children exposed to drug-resistant TB, which include adolescents and younger children. V-QUIN is comparing six months of levofloxacin to placebo for the prevention of TB among household contacts 15 years or older of people with drug-resistant TB. Like V-QUIN, TB-CHAMP is comparing six months of levofloxacin to placebo, but is focused on children below 5 years of age. ACTG A5300/IMPAACT P2003 (PHOENIx) is evaluating six months of once-daily delamanid vs. six months of isoniazid for TPT in high-risk adult, adolescent, and child contacts of people with multidrug-resistant TB.

Household and community-focused efforts to implement shorter preventive regimens for children and adolescents has the potential to improve TPT scale-up and treatment completion, which is imperative to reaching the TB prevention goals for 2022 set out in the UN General Assembly High Level Meeting for TB in 2018 [11]. A people- and family-centered approach integrated as part of frontline community-based health systems is essential for efficient and successful TPT programming and is one of the models of care recommended in the latest WHO guidance [12]. Simple tests that can be delivered at the point of care to distinguish TB infection from TB disease, and tests capable of predicting which children and adolescents with TB infection have the highest risk of progressing to active TB disease (e.g., blood-based RNA tests and blood-based tests measuring immune response) would help better prioritize and target the provision of TPT to children and adolescents.

### 2.2. TB Vaccines

In 2020, the bacillus Calmette–Guérin vaccine (BCG) “celebrated” its 100-year anniversary. The WHO’s first Expanded Program on Immunization (EPI) in 1976 included BCG and since then it has been given to more than 4 billion people. BCG is included as part of the national vaccination schedule in 154 countries, typically given to newborns, with coverage rates of >85% [13,14]. However, BCG is an imperfect vaccine that offers 60–80% protective efficacy against severe forms of tuberculosis (including TB meningitis and miliary TB) in children, prevents approximately 20% of children from TB infection, and of children infected, protects 50% from developing TB disease. It has minimal protection for adolescents and adults [15]. It has undeniably saved millions of children’s lives, both directly from the severest forms of TB as well as due to the heterologous protection that BCG gives to infants against non-TB infectious disease [16], but the protection offered by BCG isn’t adequate and the need for new options is clear [17].

The WHO has developed preferred product characteristics (PPC) for the various TB vaccination approaches both for improving the current TB vaccinations in infants aiming for safer more effective vaccines than the current BCG, as well as for vaccines for adolescents and adults who may or may not already be infected with *Mycobacterium tuberculosis* (*Mtb*). The vaccine likely to have the most immediate impact on the TB epidemic is one that can be given to adult and adolescents to prevent TB disease and as such decrease community transmission of TB. The preferred product characteristics for this vaccine include efficacy above 50% with long-lasting protection, with infrequent or no booster vaccinations required [18].

There are currently 14 candidates in clinical development (phase 1 and beyond). The TB vaccine pipeline includes whole-cell vaccines, adjuvanted proteins, and recombinant subunit vector vaccines [19]. Candidates are being investigated for the role they could play in the prevention of disease (POD), prevention of infection (POI), and prevention of recurrence (POR).

There are three candidates in phase 3 development—VPM1002, MIP, and MTBVAC. VPM1002 and MIP are being studied in a phase 3 trial to evaluate their efficacy and safety for POD among healthy household contacts (HHC) >6 years old, of newly diagnosed sputum-positive pulmonary TB patients [20]. VPM1002 is additionally being evaluated in a phase 3 study for POI among newborn infants [21]. The phase 3 POD study of VPM1002 and MIP is expected to be completed in June 2022 and the phase 3 POI study of VPM1002 is expected to be completed in July 2023. The other candidate already in phase 3 development, MTBVAC, is being evaluated for POD among infants [22]. M72/AS01E and GamTBvac, both POD candidates, do not have any pediatric or adolescent populations included in their phase 3 trials, which is a missed opportunity. The progress of all these trials and those of candidates earlier in the pipeline, including the phase 2b study to evaluate the efficacy, safety, and immunogenicity of BCG revaccination in healthy adolescents for the POI, have been considerably delayed by the lack of funding for TB R&D [1]. Given the urgent need for a new TB vaccine and the role it can play in achieving the 2022 and 2030 targets for ending TB, the funding gaps observed for TB vaccine research must urgently be filled so that the pipeline can be further diversified and so that candidates already in clinical development can be accelerated through phase 3 and to registration (if they prove to be effective and safe).

While these vaccine candidates are being developed, considerations for the rapid implementation and scale up of any new TB vaccine in infant, pediatric, and adolescent populations need to start, including how it will fit into existing pediatric and adolescent immunization programs [23] (see Table 2). A POI candidate that can be given at birth, similar to the current BCG vaccine, could be the ideal candidate as there are minimal vaccines given in adolescence. However, given the potential impact of a POD vaccine given to older children, adolescents, and adults, population-based campaigns, similar to those implemented for HPV and happening now for COVID-19 vaccinations, could be considered for the initial roll out. Candidates that are being investigated for both POD and POI offer a comprehensive approach to not only protecting infants from birth but also protecting older children and adolescents from developing TB disease if they have already been exposed to TB.

The WHO PPC outlines the importance of safe and effective vaccines and the need to include key populations such as infants, neonates, children, and adolescents, including those living with HIV, in TB vaccines R&D. Another important consideration in preparing for new vaccine candidates is to ensure that they are affordable and available to all. We have seen with COVID-19 vaccination access that a vaccine that is not available to all those who need it will not fulfil its potential and not be able to turn the tide on the TB epidemic. 

## 3. Diagnosis

TB diagnosis in children is challenging for multiple reasons. Presumption of TB is frequently limited and delayed due to the clinical evolution of TB in children and non-specific signs and symptoms. Household contact tracing and evaluation is sub-optimally implemented in most settings with missed opportunities to identify children at risk of or already sick from TB. Furthermore, bacteriological confirmation of the diagnosis in young children is difficult because TB disease in children is generally pauci-bacillary, sputum is hard for young children to produce spontaneously, non-sputum specimens are hard to collect (especially at peripheral levels of the health care system), and TB program resources and capacity are often limited given underinvestment in pediatric TB by most national TB programs. Additionally, existing TB diagnostic tools have been primarily designed and studied for use in adults, leading to sub-optimal sensitivity in children. TB diagnosis in children is most often a clinical diagnosis, with bacteriological confirmation achieved in the minority (<30%).

Compared to smear microscopy, rapid molecular tests such as GeneXpert MTB/RIF and MTB/RIF Ultra have increased the diagnosis of bacteriologically confirmed TB in children, including rifampicin-resistant TB (RR-TB). GeneXpert can be used to test for TB in sputum and other samples such as stool, nasopharyngeal, gastric and lymph node aspirates, and cerebrospinal fluid, and is recommended as the initial diagnostic test for TB and the detection of rifampicin resistance, including in children aged below 10 years with signs and symptoms of pulmonary TB. A review of 43 studies with more than 6000 participants from 21 countries showed that the sensitivity of Xpert MTB/RIF tests depends on the sample type, with gastric aspirate specimens offering the highest sensitivity (73%), followed by sputum (65%), stool (61%), and nasopharyngeal specimens with the lowest sensitivity (46%). The specificity was high in all sample types (99.8–100%). The sensitivity of Xpert MTB/RIF Ultra tests, initially recommended for use on sputum and nasopharyngeal aspirates and now also recommended for use on gastric aspirate and stool specimens, is even higher, especially in populations with paucibacillary TB, such as young children and people living with HIV [24,25].

Another rapid molecular test more recently added to the list of WHO-recommended TB diagnostic tools for TB and rifampicin-resistant/ multidrug-resistant TB (RR/MDR-TB) is Truenat MTB and Truenat MTB Plus, intended for use at the smear microscopy center level. Based on extrapolations from adult data, in children with signs and symptoms of pulmonary TB, Truenat MTB and MTB Plus may be used on sputum specimens as an initial diagnostic test for TB and rifampicin resistance. Due to the lack of available data regarding its accuracy on non-sputum samples, Truenat tests are not recommended on samples other than sputum in children [26].

Lipoarabinomannan (LAM) is a TB biomarker that can be detected in urine, which is a relatively easily accessible sample in children. In children, the only TB LAM urine test currently available and recommended by the WHO (Determine TB LAM Ag, Abbott Laboratories, Chicago, Illinois, USA) has lower sensitivity and specificity compared to Xpert on sputum or gastric aspirates, but higher sensitivity among children with severe immunosuppression [27]. TB LAM is recommended by the WHO to support TB diagnosis among adolescents and children living with HIV with signs and symptoms of TB, advanced HIV disease or serious illness, or independent of TB signs and symptoms, that have a CD4 cell count less than 200 cells/mm^3^ in inpatient settings or less than 100 cells/mm^3^ in outpatient settings [28]. Great hope has been put on the next-generation urine LAM tests, which are expected to have improved sensitivity among adult and pediatric populations regardless of HIV status, maintaining high accuracy along with the characteristics of a simple and rapid non-sputum point-of-care (POC) test [29]. The Fujifilm SILVAMP TB Lipoarabinomannan (FujiLAM, Tokyo, Japan) test has shown similar sensitivity to the Abbott Determine TB LAM test, but its specificity is substantially higher (92% vs 66%), especially in children two years or older (96% vs. 72%), making FujiLAM a possible good tool to “rule-in” children with a high pretest probability of TB, especially those hospitalized with HIV or malnutrition [30].

There are a number of other TB diagnostic tests and sampling techniques at earlier stages of development that may be even more sensitive and specific in children and better suited for use closer to the point of care. These include tongue swabs on Xpert MTB/RIF Ultra, LumiraDx (a small, battery-operated molecular and immunoassay instrument that can produce results in 20 min at the community level), and blood-based RNA and immune response tests [31].

Screening tests for TB are also needed to identify children who should receive TPT or further evaluation and diagnostic testing for TB disease [32]. Chest X-ray, symptom screening, or WHO-recommended rapid molecular tests (alone or in combination) may be used to screen for TB disease in adults and adolescents (15 years and older). Among adults and adolescents living with HIV, C-reactive protein may also be used to screen for TB disease. While in individuals younger than 15 years old who are close contacts of someone with TB, systematic screening for TB disease should be conducted using a symptom screen including any one of cough, fever or poor weight gain, or chest radiography, or both [33]. Some barriers to using radiology are its limited availability at the peripheral level of care, and limited reading capacity, especially in some settings. Great opportunities exist to optimize pediatric disease detection with computer-aided detection (CAD) technologies, which are newly recommended as an alternative to human interpretation of digital chest X-ray for screening and triage for TB—but now only for people aged 15 years or older, as CAD technologies have so far only been studied and evaluated in this age group [33]. The WHO will soon review a new class of more specific antigen-based skin tests, which may play a future role in screening children for TB as they are expected to have similar specificity to IGRAs (interferon-gamma release assays), but may be more feasible and affordable to implement and with more reliable results in children [34].

Considering all the constraints described, clinical treatment decision algorithms will continue to have an important role in supporting the diagnosis of pulmonary TB in children. These are recommended in children below 10 years old with presumptive pulmonary TB. However, the choice of the treatment decision algorithm depends on the specific population and on the availability of diagnostic tests in a given setting [25]. Finally, there are limited emerging data on the feasibility of using point-of-care lung ultrasound to support childhood TB diagnosis, but additional research is required [35,36].

## 4. Treatment

The last few years have resulted in significant changes to the medicines and regimens recommended by the WHO for the treatment of drug-resistant and drug-sensitive forms of TB. For MDR-TB, 9–12 month, all-oral, bedaquiline-based regimens have replaced 18–20 month, injectable-containing regimens [37]. The elimination of injectable agents has made treatment regimens for drug-resistant TB more tolerable and safe, eliminating the pain of daily injections, the need for hospital admission, and the risk of disability from hearing loss. Positive results from two recent trials, NEXT (6–9-months of bedaquiline, linezolid, levofloxacin, terizidone (very similar to cycloserine), ethionamide or high dose isoniazid, and pyrazinamide) and TB-PRACTECAL (6–9 months of bedaquiline, pretomanid, linezolid, and moxifloxacin), may lead the WHO to recommend even shorter all-oral treatment regimens for MDR-TB soon [38]. For MDR-TB with additional fluoroquinolone resistance (i.e., pre-extensively drug-resistant TB, pre-XDR-TB, according to 2021 definitions), the Nix-TB regimen (a 6–9-month regimen composed of bedaquiline, pretomanid, and high dose linezolid) is recommended by the WHO for use under conditions of operational research, and may be expanded to program conditions following recent results from ZeNix (a study to optimize the linezolid dose and duration in the Nix-TB regimen) [38].

Based on emerging data from IMPAACT P1108, Janssen C211, and Otsuka 232/233, children can now benefit from access to the all-oral regimens currently recommended by the WHO for RR-/MDR-TB (see Table 3). Bedaquiline and delamanid are now recommended for use in all children (previously limited to children above five and three years old, respectively) [25]. Janssen’s 20 mg bedaquiline dispersible tablet and Otsuka’s 25 mg delamanid dispersible tablet are both stringent regulatory authority (SRA) approved and available via the Stop TB Partnership Global Drug Facility [39,40]. For the pretomanid-containing regimens (Nix-TB and TB-PRACTECAL), it will still be several years before the necessary pediatric PK and safety data are available to inform its use in children. The IMPAACT network is planning P2034, a PK and safety study of a single dose of pretomanid in children receiving treatment for drug-resistant TB (planned to open 2022). A pediatric extended dosing study of pretomanid, necessary to inform the dosing and safety of pretomanid-containing regimens in children, can only be initiated once a reproductive safety study is completed in male adults (PaSEM; NCT04179500; opened September 2021; expected to complete by April 2023) [41].

For drug-sensitive TB, positive results from TBTC Study 31/ ACTG A5349 (S31/A5349) have positioned a new four-month, rifapentine- and moxifloxacin-containing regimen as an alternative to the existing six-month standard of care for adults and adolescents [42]. These results have also created a new rifapentine PK and safety data gap in children; in the S31/A5349 regimen, rifapentine is administered 1200 mg daily instead of 600 mg daily or 900 mg weekly for TPT. A PK and safety study in children is currently in development (TBTC, RADIANT-KIDS). Positive results from a pediatric phase 3 study, the SHINE trial, have enabled children with non-severe TB to share in the benefits of shorter treatment albeit different from the S31/A5349 regimen. Using routine medicines and doses, widely available in existing pediatric formulations, the SHINE trial demonstrated that children with non-severe TB who received two months of HRZ(E) followed by two months of HR did as well as children who received the six-month standard of care (two months of HRZ(E) followed by four months of HR) [43]. Furthermore, based on a systematic review and meta-analysis, the WHO recently recommended that a 6-month intensive regimen for the treatment of TB meningitis in children (6HRZEto: six months of higher dose isoniazid and rifampicin given with pyrazinamide and ethionamide) may be used as an alternative option to the 12-month regimen composed of 2HRZE/10HR recommended by the WHO since 2010 [25]. Dose-optimized, twelve- and six-month regimens that include levofloxacin are being evaluated in the recently completed TBM-KIDS trial (NCT02958709; 2HRZLfx/10HR) and the ongoing SURE trial (ISRCTN40829906; 6 HRZLfx) [44,45].

There are also 15 new drugs in clinical development, including nine from a new class or with a new mechanism of action, and a series of new collaborative initiatives to advance them as part of novel regimens [38]. Several of these new drugs are already in phase II, when experts have agreed pediatric investigational planning should begin [46] There were 6–13-year gaps observed between when delamanid, rifapentine, bedaquiline, and pretomanid were licensed for adults versus when they were or will be licensed for children. To avoid similar gaps for the next wave of new TB drugs, it is critically important that the sponsors of these products start seriously planning for their investigations in children, in collaboration with the global childhood TB community, and that these studies are designed in a way that supports expeditious completion [46].

## 5. Conclusions

It will be many years before children are able to share fully in many of the benefits of scientific progress against TB recently realized for adults. The biggest research and development gaps for children remain sensitive, non-sputum-based point-of-care diagnostics and effective TB vaccines (see Table 4).

A majority of ongoing and planned TB diagnostics and vaccines research is focused on adults and adolescents, which, along with inadequate funding, is a key barrier to advancing new and better TB diagnostics and vaccines for children. Mirroring the trend observed for overall investments in TB vaccines and diagnostics research, annual investments in pediatric TB vaccines and diagnostics research are consistently lower than those made in pediatric TB treatment research, reaching just USD 13 million and USD 20 million, respectively, in 2020 (compared to USD 24 million invested in pediatric TB drug research) [1].

The U.S. Agency for International Development (USAID), the U.S. National Institutes of Health (NIH), and the European & Developing Countries Clinical Trials Partnership (EDCTP) were the three highest funders of pediatric TB R&D in 2020. The Gates Foundation is noticeably absent from the list of funders of pediatric TB R&D, especially given its position as the largest philanthropic funder and second largest overall funder of TB R&D.

Opportunities to increase investments and accelerate pediatric TB R&D include (1) doubling down on advocacy targeting public funders (especially in G20 countries), which currently account for 71 percent of total investments in pediatric TB R&D; (2) targeting philanthropic TB research funders, which currently collectively give less than 1% of their TB R&D investments to pediatrics; and (3) honing in on multilateral funders of TB R&D other than Unitaid, which accounted for nearly all (99.8%) investments in pediatric research by multilateral organizations in 2020. COVID-19 has taught us that R&D can be expedited and money mobilized by donors and governments when a pathogen is perceived to be life-threatening and where health needs are urgent, impacting country and global economies and security, as is the case for TB, especially in low- and middle-income countries.

We need to close the R&D gaps between adults and children to ensure that everyone benefits from the latest scientific advancements [47]. We need to optimize collaboration and transparent knowledge sharing between stakeholders, enhance funding from both the public and private sectors, and ramp up investments in pediatric TB-specific basic science, vaccine, diagnostic, and implementation research, especially to help inform the introduction of pediatric TB innovations, including resource needs and other important factors such as feasibility, acceptability, and equitable access of the final products. We need to invest USD 200 million annually in capable research teams, sites, and leadership in high-burden countries in the Global South to advance science capable of ending further decades of child death and suffering from TB.

## Figures and Tables

**Table 1:** Timeline of key pediatric TB R&D milestones.

Year	Preventive Treatment	Vaccines	Diagnostics	Treatment
2010				WHO recommended revisions to pediatric dosing for first-line medicines
2011				
2012				
2013			WHO endorsed Xpert MTB/RIF as initial test in children (strong: MDR- or HIV-associated TB; conditional: all children)	Pediatric investigations of delamanid initiated
2014	FDA expanded approved indication for rifapentine to children (2+ years)			
2015			WHO endorsed LF-LAM for testing HIV-positive children with signs and symptoms of TB	
2016	Pediatric investigation of levofloxacin initiated			Pediatric investigation of bedaquiline initiated; Pediatric formulation of pyrazinamide approved by WHO Pre-Qualification Program
2017				New pediatric fixed-dose combinations aligned with 2010 WHO dosing guideline revision approved by WHO Pre-Qualification Program; Pediatric formulation of ethionamide approved by WHO Pre-Qualification Program
2018				Pediatric formulations of ethambutol, levofloxacin, moxifloxacin, cycloserine approved by WHO Pre-Qualification Program
2019	Pediatric investigation of rifapentine (including children less than 2 years old and children living with HIV) initiated Pediatric investigation of delamanid initiated	Ph 3 study of Mycobacterium Indicus Pranii (MIP) and VPM1002 (6+ years old) initiated	WHO expanded LF-LAM recommendations to include HIV-positive adolescents and children that are seriously ill, have advanced HIV disease, or a CD4 count less than 200 (for inpatients) or 100 (for outpatients)	Pediatric formulation of isoniazid approved by WHO-Pre-Qualification Program
2020		Ph 3 study of VPM1002 (infants) initiated	WHO endorsed Xpert MTB/RIF as initial test in children (strong: all) + expanded sample types to include gastric aspirate (GA), nasopharyngeal aspirate (NPA), stool WHO endorsed Xpert Ultra as initial test in children (strong: all) but limited sample types to sputum or NPA WHO endorsed Truenat MTB or MTB Plus as initial test in children (conditional)	Pediatric investigations of DLM completed FDA approved pediatric BDQ formulation Pediatric formulation of clofazimine approved by WHO Pre-Qualification Program
2021			WHO endorsed expanding pediatric sample types for use on Xpert Ultra as initial test in children to include GA and stool samples	EMA approved pediatric delamanid formulation

**Table 2:** Pediatric tuberculosis prevention research.

Study Name	Intervention	Population	Sponsor	Status
TB Preventive Treatment (TPT)				
TBTC Study 35 NCT03730181	Phase 1/2 trial evaluating the PK and safety of 3HP	Children 0–12 years old with and without HIV	CDC TBTC	Opened October 2019 Enrolling Expected completion: 2023
DOLPHIN Kids	Phase 1/2 trial evaluating the PK and safety of 3HP with DTG-based ART	Adolescents and children with HIV 4 weeks-18 years old on DTG-based ART	Unitaid via IMPAACT4TB	Planned
IMPAACT P2024	Phase 1/2 trial evaluating the PK and safety of 1HP, including when given with DTG-based ART	Children 2–13 years old with and without HIV	NIH via IMPAACT	Planned
V-QUIN ACTRN12616000215426	Phase 3 trial evaluating the safety and efficacy of 6 months of levofloxacin vs. placebo	Adult and adolescent household contacts of people with MDR-TB 15 years and older	Australian NHMRC, Government of Vietnam	Opened March 2016 Fully enrolled Expected completion: 2022
TB CHAMP ISRCTN92634082	Phase 3 trial evaluating the safety and efficacy of 6 months of levofloxacin vs. placebo	Child household contacts <5 years of age of people with MDR-TB	Unitaid, South African MRC, Wellcome Trust, British MRC	Opened January 2016 Enrolling Expected completion: 2023
PHOENIx NCT03568383	Phase 3 trial evaluating the safety and efficacy of 6 months of delamanid vs. isoniazid	Adult, adolescent, and child household contacts of people with MDR-TB	NIH via ACTG and IMPAACT	Opened June 2019 Enrolling Expected completion: 2025
TB Vaccines				
CTRI/2019/01/017026	Phase 3 trial evaluating the efficacy, safety, and immunogenicity of MIP and VPM1002 (vs. placebo) in preventing TB disease. Secondary objectives include efficacy evaluation for preventing TB infection	HHC (≥6 years old, HIV negative) of people with TB	Indian Council of Medical Research (ICMR)	Opened January 2019 Expected completion: 2022
NCT04351685	Phase 3 trial evaluating the efficacy, safety, and immunogenicity of VPM1002 (vs. BCG) in preventing TB infection.	Newborn infants (HIV-exposed and uninfected eligible)	Serum Institute of India Pvt. Ltd.	Opened November 2020 Expected completion: July 2023
NCT04975178	Phase 3 trial to evaluate the efficacy, safety, and immunogenicity of MTBVAC (vs. BCG)	HIV unexposed and HIV-exposed infants	Biofabri	Planned
NCT04152161	Phase 2b study to evaluate the efficacy, safety, and immunogenicity of BCG revaccination (vs. placebo)	BCG-vaccinated, MTB-uninfected adolescents	Bill & Melinda Gates Medical Research Institute	Opened October 2019 Expected completion: April 2023

**Table 3:** Pediatric TB treatment research.

Study Name	Intervention	Population	Sponsor	Status
Janssen C211 NCT02354014	Phase 2 trial evaluating the PK and safety of bedaquiline	HIV-negative and -positive adolescents and children 0 < 18 years old with MDR-TB	Janssen	Opened May 2016 Enrolling Expected completion: 2025
IMPAACT P1108 NCT02906007	Phase 1/2 trial evaluating the PK and safety of bedaquiline	HIV-positive and -negative adolescents and children 0 < 18 years old with RR-TB	NIH via IMPAACT	Opened August 2017 Enrolling Expected completion: 2023
Otsuka 232/233 NCT01856634/ NCT01859923	Phase 1/2 trial evaluating the PK and safety of delamanid	HIV-negative adolescents and children 0 < 18 years old with MDR-TB	Otsuka	Opened June 2013 Completed January 2020
IMPAACT P2005 NCT03141060	Phase 1/2 trial evaluating the PK and safety of delamanid	HIV-positive and -negative adolescents and children 0 < 18 years old with MDR-TB	NIH via IMPAACT	Opened January 2018 Enrolling Expected completion: 2022
IMPAACT P2034	Phase 1 trial evaluating the PK of a single dose of pretomanid	HIV-positive and -negative adolescents and children 0 < 18 years old with MDR-TB	NIH via IMPAACT	Planned Expected to open: 2022

**Table 4:** Key short-term pediatric TB research gaps.

Research Area	Research Gap(s)
TB preventive treatment	Tests to distinguish TB infection from TB disease, e.g., blood-based RNA tests, blood-based immune response tests, CAD and portable X-ray technologies Tests to predict risk of progression from TB infection to TB disease, e.g., blood-based host response tests Ultrashort child friendly TPT regimens for child contacts of people with drug-susceptible and drug-resistant TB
TB vaccines	Pediatric investigations of TB vaccine candidate M72/AS01E
TB diagnostics	Non-invasive, non-sputum, point-of-care diagnostic tests, e.g., oral swab-based rapid molecular tests for TB detection, next generation LAM tests, etc.
TB treatment	Pediatric investigation of high dose rifapentine Pediatric investigation of pretomanid (multi-dose) Non-sputum tests to facilitate treatment monitoring and shortening, e.g., blood-based host response tests

## Data Availability

Not applicable.
